# Physical, Chemical and Proteomic Evidence of Potato Suberin Degradation by the Plant Pathogenic Bacterium *Streptomyces scabiei*

**DOI:** 10.1264/jsme2.ME16110

**Published:** 2016-11-17

**Authors:** Carole Beaulieu, Amadou Sidibé, Raoudha Jabloune, Anne-Marie Simao-Beaunoir, Sylvain Lerat, Ernest Monga, Mark A. Bernards

**Affiliations:** 1 Centre SÈVE, Département de Biologie, Université de SherbrookeSherbrooke (QC), J1K 2R1Canada; 2 Département de Mathématiques, Université de SherbrookeSherbrooke (QC), J1K 2R1Canada; 3 Department of Biology, University of Western OntarioLondon (ON), N6A 5B7Canada

**Keywords:** common scab, degradation, proteomics, *Streptomyces scabies*, suberin

## Abstract

Potato peels consist of a tissue called phellem, which is formed by suberized cell layers. The degradation of suberin, a lipidic and recalcitrant polymer, is an ecological process attributed to soil fungal populations; however, previous studies have suggested that *Streptomyces scabiei*, the causal agent of potato common scab, possesses the ability to degrade suberin. In the present study, *S. scabiei* was grown in medium containing suberin-enriched potato phellem as the sole carbon source and its secretome was analyzed periodically (10- to 60-d-old cultures) with a special focus on proteins potentially involved in cell wall degradation. Although the amount and diversity of proteins linked to polysaccharide degradation remained high throughout the experiment, their abundance decreased over time. In contrast, proteins dedicated to lipid metabolism represented a small fraction of the secretome; however, their abundance increased during the experiment. The lipolytic enzymes detected may be involved in the degradation of the aliphatic fraction of suberin because the results of optical and transmission electron microscopy examinations revealed a loss in the integrity of suberized tissues exposed to *S. scabiei* cells. Chemical analyses identified a time period in which the concentration of aliphatic compounds in potato phellem decreased and the sugar concentration increased; at the end of the 60-d incubation period, the sugar concentration in potato phellem was significantly reduced. This study demonstrated the ability of *S. scabiei* to degrade the aliphatic portion of suberin.

The potato periderm consists of a physical barrier that protects tubers against microbial infections as well as dehydration. The potato periderm is formed of three cell types: phellem, phellogen, and phelloderm. The skin tuber, the outer part of the periderm, consists of layers of phellem cells. The active suberization of phellem cells during tuber formation leads to the death of phellem cells. Suberization is responsible for the impermeable nature of the protective skin. When observed under transmission electron microscopy, suberized cell walls appear lamellate; light lamellae alternating with opaque ones (8). Suberin is a complex polyester composed of a large proportion of long-chain α,ω-diacids and ω-hydroxyacids and of smaller amounts of long-chain fatty acids and fatty alcohols (4). These aliphatic compounds are linked to a lignin-like structure through esterification to ferulic acid or glycerol (8). While some authors use the term suberin to exclusively design the lipidic polyester (8), suberin is defined here as a polymer comprising polyaliphatic and polyaromatic domains (4).

Suberin is a recalcitrant biopolymer because it is slowly biodegraded in soil (9). Although the degradation of suberin has not been extensively examined, it has been attributed to soil fungal populations (14). When *Aspergillus nidulans* was grown on suberized cell walls, the degradation of the suberin aromatic domain was corroborated by the identification of some degradation products, whereas the aliphatic domain remained virtually intact (20). However, a whole-genome transcriptome analysis of *A. nidulans*, which was conducted in order to identify the main pathways involved in suberin degradation, proposed that initial suberin degradation involved ester hydrolysis, possibly through the actions of cutinase 1 and some lipases (21).

Previous studies have suggested that *Streptomyces scabiei*, the causal agent of potato common scab, possesses the ability to degrade suberin. This pathogenic bacterium has been shown to grow using suberin as the sole source of carbon (25), exhibits strong esterase activity in the presence of suberized cells (1), and its genome contains potential cutinase genes, including *sub1*, which was previously reported to be specifically induced in the presence of suberin (15). When *S. scabiei* was grown for 5 d in the presence of suberin, its secretome included several enzymes predicted to play a role in lipid metabolism; however, these enzymes accounted for a small fraction of all extracellular enzymes. The abundant production of extracellular glycosyl hydrolases has been demonstrated under the same conditions (16). As the suberin preparation was known to contain a non-negligible amount of polysaccharides (25), it could not been excluded that during a 5-d incubation period, *S. scabiei* growth on suberin strictly depended on polysaccharides. The diversity and abundance of *S. scabiei* proteins involved in sugar catabolism have been attracting interest because only a few polysaccharide-degrading enzymes (β-glucosidases and endo-1,5-α-l-arabinosidase) have been detected in the secretome of *A. nidulans* grown in the presence of suberized cells (20).

In the present study, *S. scabiei* was grown in medium containing suberin-enriched potato phellem (hereafter called potato phellem) for extended periods of time, and its secretome was periodically characterized. The integrity of suberized tissues exposed to *S. scabiei* cells was examined using transmission electron microscopy and analytical chemistry tests were employed in order to detect changes in the composition of phellem. The results of the present study provide evidence for *S. scabiei* degrading, at least partially, potato suberin.

## Materials and Methods

### Culture conditions

*S. scabiei* strain EF-35 (7) was grown in PPM medium containing potato phellem as the sole source of carbon in order to analyze the bacterial secretome during a prolonged incubation as well as morphological and chemical modifications in phellem within the incubation time. Potato phellem was obtained from potato peels through the enzymatic digestion of cell wall polysaccharides followed by Soxhlet extraction to eliminate wax components and soluble aliphatic compounds (13). PPM was composed of ground phellem (0.1%) and a mineral solution (MiS) containing (NH_4_)_2_SO_4_ (0.5 g L^−1^), K_2_HPO_4_ (0.5 g L^−1^), MgSO_4_-7H_2_O (0.2 g L^−1^), and FeSO_4_-7H_2_O (0.01 g L^−1^). A *S. scabiei* inoculum was prepared as described by Komeil *et al.* (16). The bacterium was grown with shaking (250 rpm) at 30°C.

In the secretome analysis, *S. scabiei* was grown for 60 d and periodically (after 10, 20, and 30 d of incubation), the PPM culture was centrifuged (20 min at 3,450×*g*), and two thirds of the volume of the supernatant was kept for the proteomic analysis. The pellet containing bacteria and insoluble phellem was resuspended in the remaining volume of the supernatant in order to avoid the breakdown of possible protein cascades and a volume of MiS equivalent to the sampled volume of the supernatant. The incubation was then resumed under the same conditions. The culture supernatant was also sampled at the end of the 60-d incubation period. EDTA (0.03% [w/v]) was added to each supernatant sample in order to prevent protein degradation. Proteins were concentrated by filtration (Amicon Ultra-15 Centrifugal Filters 10-K) before the proteomic analysis as described by Komeil *et al.* (16). The experiment was performed in duplicate.

In the chemical characterization of phellem, *S. scabiei* was incubated for a period of 0 to 60 d, as described above. At the end of each incubation period, cultures were centrifuged (20 min at 3,450×*g*). The pellets containing suberin-enriched phellem and mycelia were resuspended in 20 mL of sterile water and autoclaved for at 121°C for 15 min. Sterilized phellem residues were then washed three times in sterile water to eliminate bacterial cell debris and finally air-dried at 50°C for 24 h. The experiment was performed in triplicate for each incubation time.

### Visualization of suberin degradation

An inoculum of *S. scabiei* (100 μL) prepared as described by Komeil *et al.* (16) and eight disks of potato phellem with a diameter of 8 cm were added to 25 mL of MiS. The culture was incubated with shaking for 12 months. Fresh MiS (5 mL) was added to the culture every month and a fresh inoculum every 3 months. In control flasks, the *S. scabiei* inoculum was omitted. The experiment was performed in triplicate. Control and treated disks were photographed at the end of the experiment.

Suberin-enriched phellem that had been incubated in the presence of *S. scabiei* for 0, 5, 20, 30, and 60 d was fixed in 2.5% (v/v) glutaraldehyde in 0.1 M phosphate buffer (pH 7.0). Samples were post-fixed in 1% (w/v) osmium tetroxide in the same buffer. They were dehydrated through a series of graded ethanol (for 5–15 min at each concentration): 70%, 85%, 95%, and 100% three times; and treated three times with propylene oxide. Samples were then infiltrated and coated with Spurr resin and cut into ultrathin sections. Coloration was performed with lead citrate and uranyl acetate. Samples were observed using the transmission electron microscope Hitachi H-7500 at 80 kV.

### Aliphatic and sugar content measurements in the suberin-enriched potato periderm

Aliphatic suberin monomers were extracted from phellem as proposed by Browse *et al.* (6) and later modified by Meyer *et al.* (22). Methyl ester/TMS ether derivatives from insoluble polyaliphatic monomer fractions were quantified on a Varian CP-3800 gas chromatograph (GC) equipped with a flame ionization detector (FID). Monomer identification was accomplished on a Varian MS 220 ion trap mass spectrometer (MS). The GC had a pair of CP-Sil 5 CB low bleed MS columns (WCOT silica 30 m×0.25 mm ID), with one column directed to the FID and the other to the MS. The temperature of the injector oven was 250°C, and the FID oven was set to 300°C. After sample injection into each column (splitless mode), monomers were eluted using the following program: 70°C held for 2 min, increased to 200°C at 40°C min^−1^ and held for 2 min, increased to 300°C at 3°C min^−1^ and held for 9.42 min, for a total run time of 50 min. High purity helium was used as the carrier gas with a flow rate of 1 mL min^−1^.

The amount of sugars in phellem samples was measured by hydrolyzing polysaccharides as described by Blakeney *et al.* (5); the released monosaccharides were then reduced and acetylated as proposed by Blakeney *et al.* (5). The alditol acetate derivatives obtained were quantified by gas chromatography on a Varian 3800 GC equipped with a flame-ionization detector (FID) and CP-Sil 88 column according to Shao *et al.* (30).

### Proteomic analysis

Supernatants were recovered and the secreted proteins were concentrated and subjected to sodium dodecyl sulfate-polyacrylamide gel electrophoresis (10% [w/v] SDS-PAGE) according to Komeil *et al.* (16). In-gel protein digestion and mass spectrometry were performed at the Proteomics Platform of the Eastern Quebec Genomics Center (Quebec City, Canada) using a quadrupole time-of-flight mass spectrometer (Qq-TOF) (AB Sciex) coupled to a HPLC as previously described (16).

All MS/MS samples were analyzed for peptide identification using Mascot (Matrix Science, London, UK; version 2.4.1). Mascot was set up to search the UR-014_2 *Streptomyces* database assuming the digestion enzyme trypsin. Mascot was searched with a fragment ion mass tolerance and parent ion tolerance of 0.1 Da. Scaffold version 4.3.2 (Proteome Software, Portland, OR) was used to validate peptide and protein identifications. Identification probability was performed using the peptide Prophet algorithm (12) and Protein Prophet algorithm (23). Identification was accepted when probability was greater than 11 and 84% for peptides and proteins, respectively, in order to achieve a false discovery rate lower than 1.0%. The validation of protein identification also required the protein to contain at least two identified peptides.

### Analysis of the secretome profile

The extracellular localization of the protein was assessed using SignalP (27), Phobius (11), SecrotomeP (2), TatP (3), and Tatfind (28) programs, while NCBI, Uniprot, KEGG, and COG resources were used to predict protein function.

The normalized spectral count (NSpC) was defined as the spectral count of a protein divided by its molecular weight. The normalized spectral abundance factor (NSAF) was the ratio between the NSpC of a protein and total NSpC.

The similarity index (*S*) of secretome profiles between two sampling dates (*a* and *b*) was assessed by the formula $S^{a,b}=1-\frac{\sum_{k=1}^{n}\left|N_{k}^{a}-N_{k}^{b}\right|}{\sum_{k=1}^{n}\text{Max}\;\left(N_{k}^{a},N_{k}^{b}\right)}$, where *N_a_* and *N_b_* are the NSpC of the same protein *k*.

## Results

### Morphological modifications in phellem during a prolonged incubation in the presence of *S. scabiei*

**Fig. 1:**
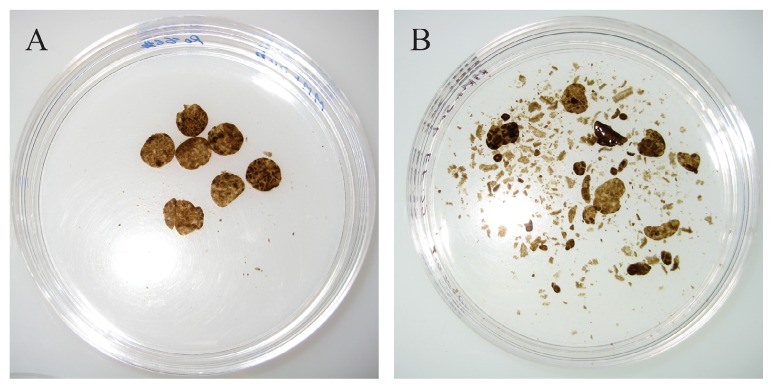
Potato phellem disks after a 12-month incubation in A) the absence or B) presence of *Streptomyces scabiei* EF-35.

After a one-year incubation period, phellem disks remained almost intact in the absence of the *S. scabiei* inoculum, indicating that no important mechanical disruption of the polymer occurred during the prolonged incubation (Fig. 1A). When *S. scabiei* EF-35 inocula were added to the medium, fractioning of the disks was observed; however, a large amount of insoluble material still remained unutilized by the bacteria (Fig. 1B).

**Fig. 2:**
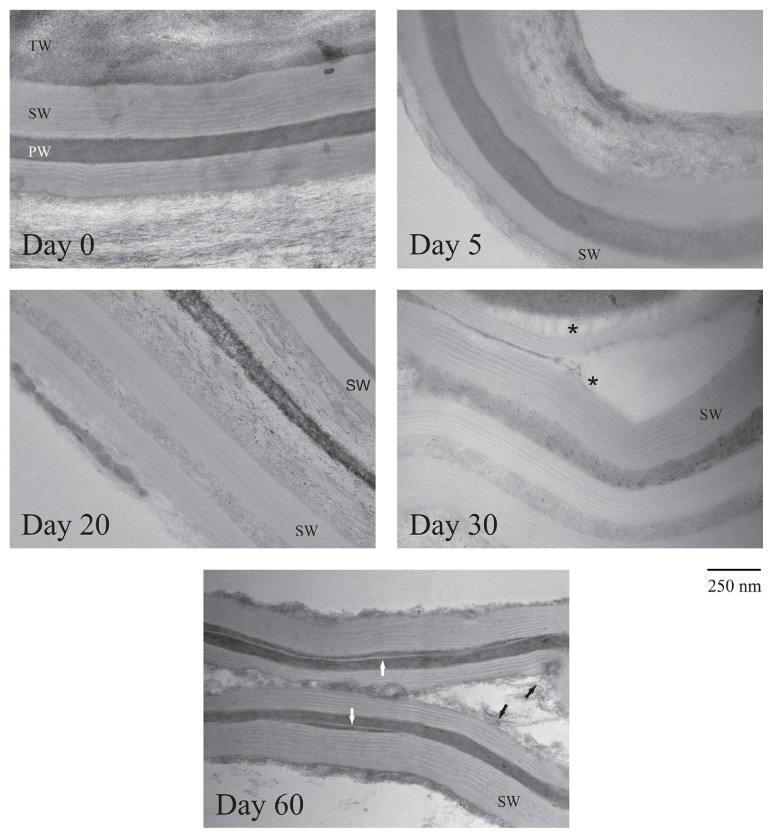
Transmission electron micrographs of potato phellem incubated in the presence of *Streptomyces scabiei* EF-35 for 0 to 60 d. PW: primary cell wall; SW: suberized secondary cell wall; TW: tertiary cell wall. ^*^ shows the zone of secondary wall detachment. White arrows show suberin lamellae detached from the secondary wall. Black arrows show broken suberin lamellae.

Ground potato phellem incubated with *S. scabiei* for up to 60 d was periodically observed using transmission electron microscopy. In the first 20 d of the incubation, there was no clear evidence of morphological modifications (Fig. 2). At day 30, detachment of the secondary suberized wall from the juxtaposed cell walls was more frequently observed. After a 60-d incubation period, clear evidence of suberin degradation was observed. Suberin lamellae became detached from the secondary wall and broken lamellae were released. The amount of polysaccharides also appeared to be markedly reduced (Fig. 2).

### Chemical modifications in potato phellem during a prolonged incubation in the presence of *S. scabiei*

**Fig. 3:**
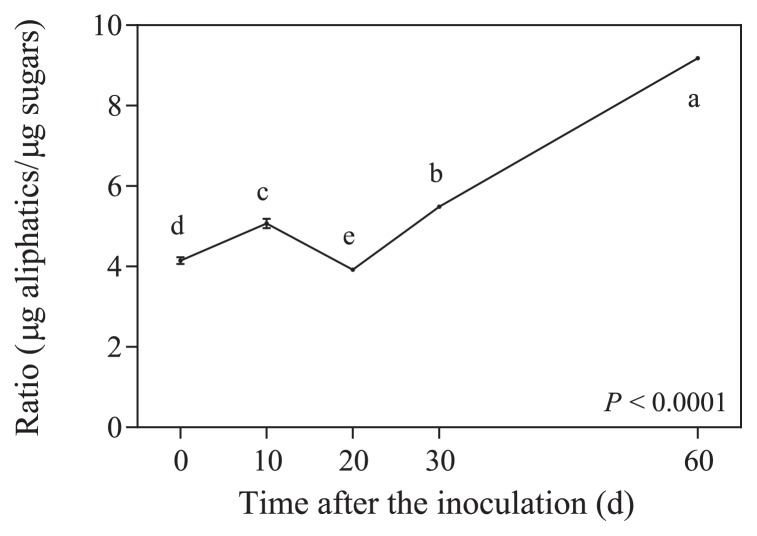
Ratio between concentrations of aliphatic compounds (fatty acids, fatty alcohols, α,ω-dioic acids, ω-hydroxy acids, and ferulic acid) and sugars (arabinose, xylose, rhamnose, fucose, mannose, galactose, and glucose) in potato phellem after an incubation in the presence of *Streptomyces scabiei* EF-35 for 0 to 60 d. Values accompanied by the same letter did not differ significantly (LSD test).

**Fig. 4:**
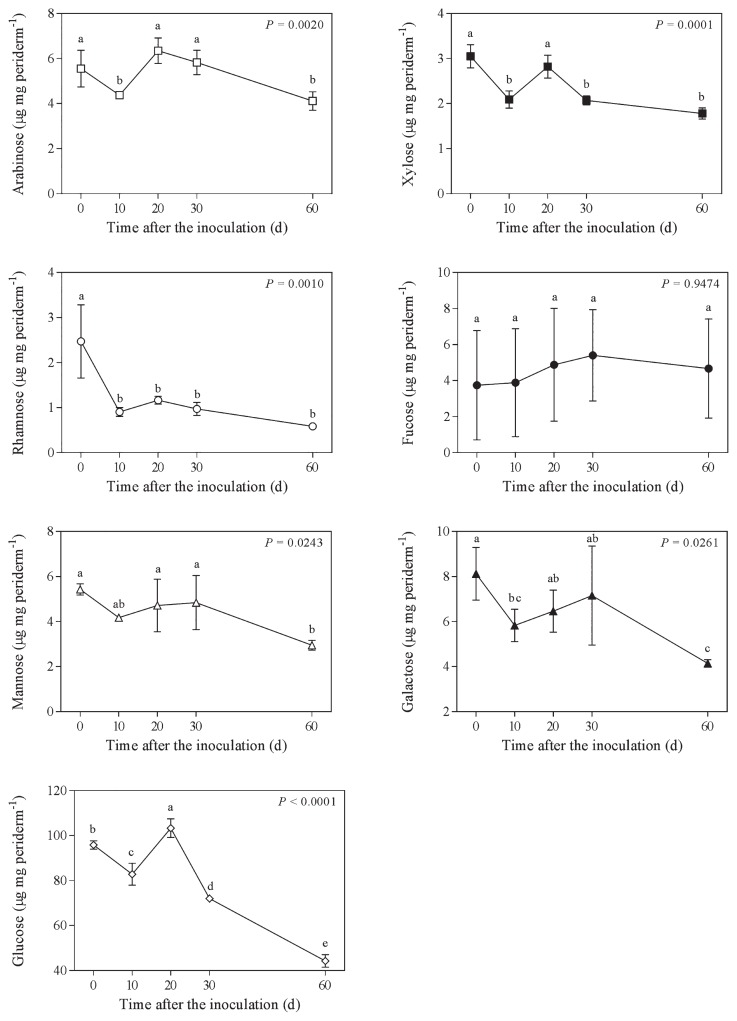
Concentration of sugars in potato phellem after an incubation in the presence of *Streptomyces scabiei* EF-35 for 0 to 60 d. Values within a panel accompanied by the same letter did not differ significantly (LSD test).

**Fig. 5:**
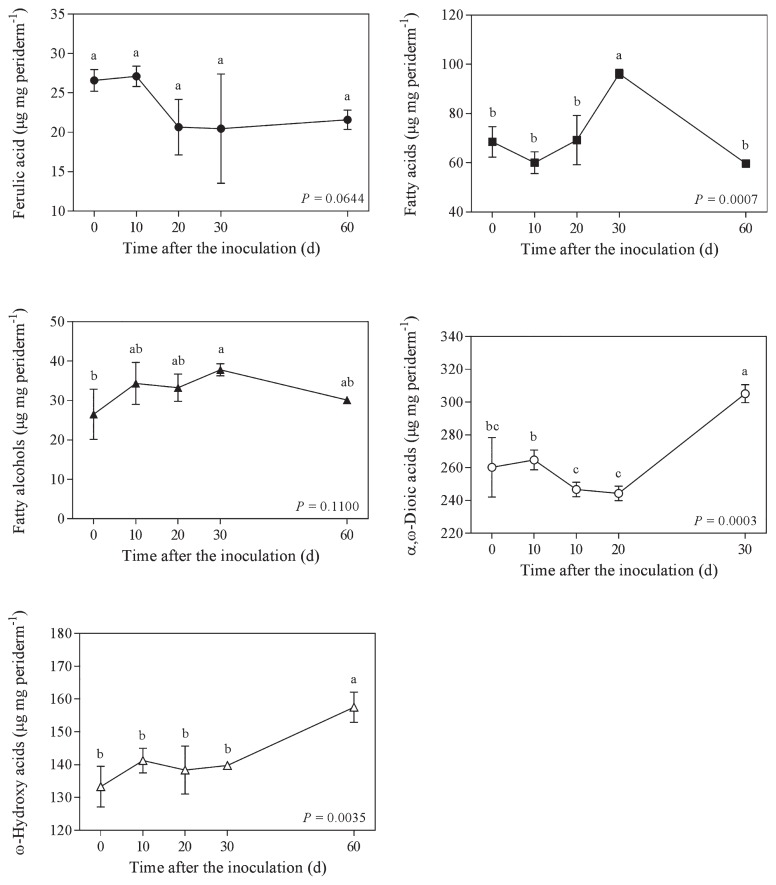
Concentration of aliphatic compounds in potato phellem after an incubation in the presence of *Streptomyces scabiei* EF-35 for 0 to 60 d. Values within a panel accompanied by the same letter did not differ significantly (LSD test).

At the beginning of the experiment, the ratio between the amount of aliphatic compounds and sugars in potato phellem was 4.15 and reached 9.18 by the end of the 60-d incubation period (Fig. 3). At the end of the incubation, the concentrations of all sugars, except fucose, were reduced (Fig. 4). The proportions of α,ω-dioic acids and ω-hydroxy acids in phellem after the 60-d incubation were significantly higher than their initial concentrations (Fig. 5). This was not the case for all aliphatic compounds because the concentrations of fatty acids decreased while ferulic acid and fatty alcohol concentrations remained stable (Fig. 5). The ratio between the amount of aliphatic compounds and sugars increased gradually over time, except between day 10 and day 20, during which it significantly decreased from 5.1 to 3.9 (Fig. 3); this period corresponded to a significant decrease in the relative concentration of α,ω-dioic acids (Fig. 5) and an increase in glucose, arabinose, and glucose concentrations (Fig. 4). Glucose was the sugar most efficiently released from phellem between day 20 and 30 of the incubation (Fig. 4).

### Effects of the incubation time on the *S. scabiei* extracellular protein profile

**Fig. 6:**
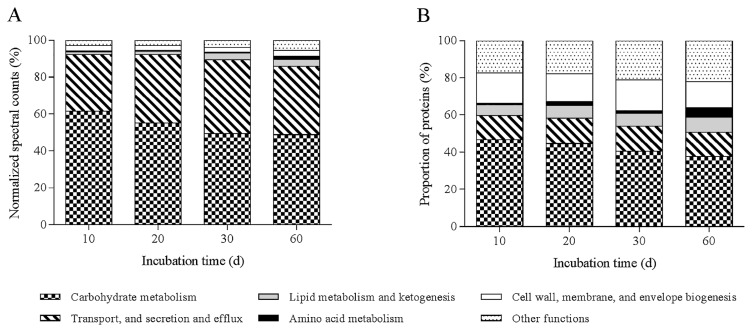
Temporal variations in *Streptomyces scabiei* protein distribution within functional groups. A) Relative protein abundance (normalized spectral abundance factor) within functional protein groups; B) relative number of proteins assigned to each functional group.

A total of 180 proteins with predicted extracellular locations were detected in *S. scabiei* cultures grown in the presence of potato phellem (day 10 to day 60) and these proteins were divided into 13 functional groups (Table S1). The number of proteins was relatively stable over time, varying from a minimum of 136 (day 60) to 146 (day 20 and 30) (Table S1). However, protein abundance was affected more by the incubation time; the maximal NSpC was obtained at day 20 (153.72) and the lowest at day 60 (89.02) (Table S1). Proteins assigned to four functional groups (Carbohydrate metabolism, Transport, secretion, and efflux, Amino acid metabolism, and Lipid metabolism) as well as proteins of unknown functions accounted for more than 1% of the total NSAF at one or more sampling dates (Fig. 6). The functional group Carbohydrate metabolism represented the most important protein category in relation to protein diversity and abundance at all sampling dates (Fig. 6). Nevertheless, the proportion of Carbohydrate metabolism proteins decreased over time, representing 61.5 and 48.9% of the total NSAF at day 10 and 60, respectively (Fig. 6). Within Carbohydrate metabolism proteins, glycosyl hydrolases were the most abundant proteins, representing more than 80% of NSpC associated with the Carbohydrate metabolism class. Carbohydrate esterases and polysaccharides lyases were poorly represented within the class and their NSAF decreased by approximatively 2.6- and 12-fold, respectively (Table S2). Five predicted cellulases (C9Z9L6, C9Z9L7, C9ZEQ0, C9ZEQ1, and C9ZEP9) emerged among the seven most abundant proteins of this class at all sampling times (Table S1).

Eleven proteins were assigned to the Lipid metabolism class (Table 1). Proteins from this group accounted for 1.4% of the total NSAF at day 10 and constantly increased to 3.7 by the end of the experimental period (Fig. 6). All proteins in this class, except C9ZG71 (esterase A), exhibited a maximal NSAF at day 30 or 60. C9ZG71 accounted for 25.5 and 0.8% of the total NSpC in the Lipid metabolism class at day 10 and 60, respectively (Table 1). C9Z707, a putative CoA-C acetyl transferase, and C9Z4H0, a putative 3-hydroxyacyl Co A reductase, were predicted to be involved in β-oxidation, a catabolic process by which fatty acids are broken down (24), and as with most proteins in the Lipid metabolism class, they exhibited higher NSAF on the last days of the incubation.

**Fig. 7:**
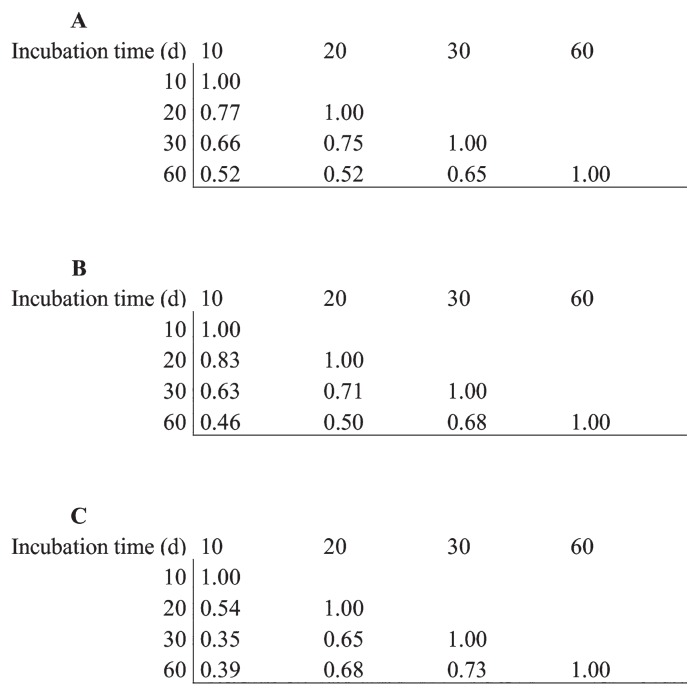
Similarity index of extracellular protein profiles between 10- to 60-d-old PPM cultures of *Streptomyces scabiei* EF-35. A) Total extracellular proteins; B) proteins associated with the Carbohydrate metabolism class only, and C) proteins associated with the Lipid metabolism class only.

Variations in protein profiles over time were estimated using a similarity index. When proteins from all functional groups were considered, the highest similarity index (0.77) was found between profiles from 10- and 20-d-old supernatants (Fig. 7A) and the lowest similarity index (0.52) was associated with profiles from day 10 and day 30 and 60. When the similarity index was measured within proteins of the Carbohydrate metabolism class only, the highest score was of 0.83 (between day 10 and 20) and the lowest was of 0.46 (between day 10 and 60) (Fig. 7B). Protein profiles of the Lipid metabolism class were the most divergent between day 10 and 30 (similarity index of 0.39) and protein profiles in this class exhibited the lowest level of variation between day 30 and 60 (similarity index of 0.73) (Fig. 7C).

## Discussion

We previously demonstrated that when grown in the presence of suberin-enriched potato phellem for 5 d, the *S. scabiei* secretome included proteins that participate in suberin degradation; however, the secretome was characterized by the abundance of glycosyl hydrolases (16). In the present study, *S. scabiei* was grown in the presence of potato phellem for extended periods of time in order to follow the kinetics of various classes of extracellular proteins and especially proteins involved in carbohydrate or lipid metabolism. This study revealed that the amount and diversity of proteins linked to polysaccharide degradation remained high, even for prolonged cultures. However, the results obtained provided evidence for the growth of *S. scabiei* on potato phellem not depending exclusively on polysaccharides because this organism undeniably possesses the ability to degrade suberin, albeit at a slow rate (as expected for a recalcitrant biopolymer (9). The fragmentation of potato phellem observed after a 1-year incubation period is unlikely due to the sole action of sugar catabolic enzymes because microscopy analyses on potato phellem exposed to *S. scabiei* revealed clear detachment and the breaking of light suberin lamellae that represent the core of the aliphatic suberin polyester (8).

A chemical analysis of potato phellem exposed to the presence of *S. scabiei* also demonstrated the ability of *S. scabiei* to degrade the aliphatic portion of suberin. Lipidic compounds were more efficiently released from suberin-enriched phellem than sugars from day 10 to day 20, as indicated by a decrease in the relative concentration of α,ω-dioic acids, the main constituents of the aliphatic fraction of potato suberin (31), and increases in the concentrations of glucose, arabinose, and xylose. Since a high similarity index was observed between the protein profiles of the Carbohydrate metabolism class at day 10 and 20, the increase observed in the sugar concentration was unlikely due to variations within proteins dedicated to carbohydrate metabolism. It is also improbable that this increase was due to the inhibition of glycosyl hydrolases because the active degradation of polysaccharides was observed later without the marked production of new enzymes in the Carbohydrate metabolism class. The low relative sugar release rate between day 10 and day 20 is still unexplained; however, we speculate that suberin-enriched phellem became temporarily recalcitrant to the actions of several carbohydrate-degrading enzymes, possibly due to the impermeable nature of suberin after the exhaustion of readily accessible sugars. The actions of some lipolytic enzymes are needed at this time period in order to allow better access of the hydrophilic glycosyl hydrolases to their enzymatic substrates.

In contrast to proteins in the Carbohydrate metabolism class that exhibited more similar profiles at the beginning of the cultures (between day 10 and 20), the profiles of proteins involved in lipid metabolism were more similar in late cultures (between day 30 and 60). The protein profiles of the Lipid metabolism class were the most divergent between 10-d-old and 30-d-old cultures, and the only period at which aliphatics were more efficiently released from potato phellem than from sugars corresponded to this time interval. It is also during this interval that Sub1, a protein sharing sequence similarity with a fungal suberinase (15), was detected in the *S. scabiei* secretome. Consequently, this protein, the presence of which in the *S. scabiei* secretome is reported here for the first time, may be of importance for the degradation of the aliphatic domain of suberin. Since lipolytic enzymes produced by *Streptomyces* species are often induced by the presence of their natural substrate (18), the detection of Sub1 after a 20-d incubation may be due to better access to the aliphatic suberin fraction after the partial depolymerization of cell wall polysaccharides remaining in the phellem preparation during the first 10 d of the incubation.

Esterases and lipases are the types of enzymes expected to degrade polyesters such as suberin (17). The abundance of most lipases and esterases detected in this study (Sub1, C9Z6Y6, C9YUN4, C9Z1F6, and C9YTK3) increased over time. The maximal production of these lipolytic enzymes in the late phase of the culture may reflect the bacterial growth phase. In some streptomycetes, esterases and lipases were found to be produced during the stationary growth phase (29). The final concentrations of ω-hydroxyacids and α,ω-dioic acids were higher than their initial concentrations, while those of other aliphatics decreased or remained stable, which indicates the preferential release of certain types of aliphatic compounds from suberin. Therefore, the lipases and esterases detected in this study may exhibit preferences for particular substrates, as has been previously reported for such enzymes (19). This finding also reveals the concomitant utilization by *S. scabiei* of sugars and lipidic compounds, as has been previously reported for other streptomycetes (26). When streptomycetes are cultivated in the presence of sugars and lipids, bacteria typically use fatty acids directly as storage products or as part of their structural membrane instead of as a carbon source (26). The detection of β-oxidation enzymes in old cultures suggests that sugar assimilation generates a low energetic yield in the late phase of a culture.

Based on the microscopy, analytical, and proteomic data presented in this study, a model for the degradation of potato phellem by *S. scabiei* has been proposed. After the exhaustion of readily accessible sugars, suberin-enriched phellem becomes recalcitrant to the actions of several carbohydrate-degrading enzymes, possibly due to the impermeable nature of suberin. Lipases and esterases may cleave ester bonds within the aliphatic domain of suberin, thereby rendering the periderm more permeable to the abundant and various hydrophilic carbohydrate-degrading enzymes produced by *S. scabiei*. However, the consequential and gradual disappearance of cell wall sugars over time may lead to the loss of certain proteins dedicated to polysaccharide degradation and to an increase in proteins involved in lipolysis, including Sub1 and proteins involved in β-oxidation.

While some studies previously provided evidence of suberin degradation by fungal species (10), this study demonstrated the ability of a bacterium to degrade the aliphatic portion of suberin. Further research to characterize *S. scabiei* proteins participating in suberin degradation is currently underway.

## Supplementary Information



## Figures and Tables

**Table 1:** *Streptomyces scabiei* extracellular proteins associated with the Lipid metabolism class

Protein	Putative function	Normalized spectral abundance factor of proteins associated with the Carbohydrate metabolism class (% within the class)			

		Day 10	Day 20	Day 30	Day 60
C9Z6Y6	Cholesterol esterase	0.54 (39.42)	0.68 (34.17)	1.20 (34.19)	1.25 (33.42)
C9Z1F6	Lipase	0.22 (16.06)	0.36 (18.09)	0.56 (15.95)	0.62 (16.58)
C9YTK3	Lipase	0.11 (8.03)	0.28 (14.01)	0.44 (12.54)	0.36 (9.63)
C9YUN4	Cholesterol esterase	0.03 (2.19)	0.10 (5.03)	0.14 (3.99)	0.15 (4.10)
C9ZCR8	Suberinase (Sub1)	0.00 (0.00)	0.14 (7.34)	0.26 (7.41)	0.37 (9.89)
C9Z5Z2	Glycerophosphoryl diester phosphodiesterase	0.10 (7.30)	0.14 (7.04)	0.18 (5.13)	0.20 (5.35)
C9ZG71	Esterase A	0.35 (25.55)	0.19 (9.55)	0.15 (4.27)	0.03 (0.80)
C9Z4H0	3-Hydroxyacyl coA reductase	0.00 (0.00)	0.05 (2.52)	0.45 (12.82)	0.67 (17.91)
C9Z707	Acetyl-coA C-acetyltransferase (FadA)	0.00 (0.00)	0.00 (0.00)	0.00 (0.00)	0.04 (1.07)
C9ZCR0	Sphingolipid ceramide N-deacylase	0.01 (0.73)	0.01 (0.50)	0.04 (1.14)	0.01 (0.27)
C9Z8A2	Phospholipase C	0.01 (0.73)	0.04 (2.01)	0.09 (2.56)	0.05 (1.34)
