# Supplementary material for: Physical, Chemical and Proteomic Evidence of Potato Suberin Degradation by the Plant Pathogenic Bacterium Streptomyces scabiei
Source: Microbes Environ. 2016 Nov 17;31(4):427–34. doi: 10.1264/jsme2.ME16110 (PMC5158115; doi:10.1264/jsme2.ME16110)
Supplement: Supplementary file 1 [file 31_427_s1.pdf]

Supplementary Table S1 Normalized spectral count (NSpC) of predicted extracellular proteins produced by *Streptomyces scabies* EF-35 in the presence of suberin-enriched potato

Protein assignation	Putative Function	Day 10		Day 20		Day 30		Day 60	
		NSpC	S.D.	NSpC	S.D.	NSpC	S.D.	NSpC	S.D.
Amino acid metabolism									
C9YTF8	serine hydroxymethyltransferase 4	0.000	0.000	0.000	0.000	0.000	0.000	0.144	0.068
C9Z8U5	zinc aminopeptidase	0.000	0.000	0.011	0.015	0.000	0.000	0.130	0.061
C9ZGF4	gamma-glutamyl transferase	0.000	0.000	0.000	0.000	0.000	0.000	0.015	0.022
C9Z443	aspartate aminotransferase	0.000	0.000	0.000	0.000	0.000	0.000	0.044	0.063
C9YTK4	phosphoserine aminotransferase	0.713	0.194	0.525	0.177	0.913	0.548	1.163	0.018
C9YY61	zinc-binding carboxypeptidase	0.000	0.000	0.005	0.007	0.000	0.000	0.005	0.007
C9ZGG7	gamma-glutamyltranspeptidase	0.000	0.000	0.000	0.000	0.033	0.023	0.041	0.035
Carbohydrate metabolism									
C9YU69	poly(3-hydroxyalkanoate) depolymerase C	0.098	0.046	0.141	0.138	0.000	0.000	0.011	0.015
C9ZE74	acetyl-xylan esterase	0.000	0.000	0.110	0.155	0.183	0.017	0.024	0.034
C9ZEC6	glycosyl hydrolase	0.005	0.007	0.010	0.000	0.005	0.007	0.005	0.007
C9YU29	beta-mannosidase	0.734	0.199	1.055	0.055	0.984	0.022	0.586	0.254
C9YVP5	cellulase B	0.756	0.054	0.795	0.145	0.513	0.000	0.449	0.018
C9Z4A0	glycosyl hydrolase	0.000	0.000	0.000	0.000	0.063	0.045	0.063	0.090
C9Z623	licheninase	1.327	0.182	1.554	0.182	1.619	0.077	1.574	0.434
C9Z725	polysaccharide lyase	0.009	0.013	0.009	0.013	0.000	0.000	0.000	0.000
C9Z885	glycosyl hydrolase	0.000	0.000	0.000	0.000	0.013	0.000	0.013	0.000

C9ZB17	glycosyl hydrolase	0.040	0.000	0.036	0.006	0.024	0.011	0.012	0.017
C9YU11	glycosyl hydrolase	0.013	0.018	0.000	0.000	0.000	0.000	0.000	0.000
C9YU66	pectate lyase	1.889	0.262	1.648	0.550	0.648	0.550	0.185	0.000
C9YW88	cellulase/xylanase	0.183	0.017	0.329	0.259	0.500	0.052	0.220	0.000
C9YX59	alpha-L-fucosidase	0.038	0.000	0.063	0.018	0.025	0.018	0.019	0.027
C9YYE6	pectate lyase	1.404	0.361	0.660	0.120	0.106	0.090	0.011	0.015
C9Z1U5	exo-alpha-sialidase	0.353	0.028	0.392	0.028	0.206	0.014	0.059	0.083
C9ZAZ8	glycosyl hydrolase	0.298	0.054	0.457	0.211	0.870	0.048	0.529	0.190
C9ZD71	carbohydrate P isomerase	0.053	0.074	0.000	0.000	0.000	0.000	0.000	0.000
C9YT62	rhamnogalacturonate lyase	0.119	0.024	0.025	0.036	0.000	0.000	0.000	0.000
C9YY84	transketolase A	0.000	0.000	0.000	0.000	0.000	0.000	0.013	0.019
C9Z1U1	GlcNAc-PI de-N-acetylase	0.000	0.000	0.000	0.000	0.000	0.000	0.019	0.026
C9Z273	cellulase	0.103	0.145	0.109	0.154	0.263	0.009	0.058	0.082
C9Z348	glycosyl hydrolase	0.000	0.000	0.000	0.000	0.013	0.019	0.000	0.000
C9ZBL8	glucosidase	0.234	0.000	0.319	0.030	0.287	0.015	0.245	0.105
C9ZD77	glycosyl hydrolase	0.090	0.014	0.040	0.000	0.010	0.014	0.000	0.000
C9ZGD5	alpha-L-fucosidase	0.014	0.019	0.000	0.000	0.000	0.000	0.000	0.000
C9ZHE7	carbohydrate debranching enzyme	0.195	0.131	0.115	0.134	0.154	0.007	0.069	0.076
C9YSR8	fucosidase	0.239	0.000	0.113	0.100	0.085	0.100	0.028	0.040
C9YSS1	alpha-galactosidase	0.052	0.049	0.026	0.012	0.000	0.000	0.000	0.000
C9YT63	beta-xylosidase	0.730	0.042	0.640	0.000	0.350	0.156	0.200	0.141
C9YTK2	cellulase	1.907	0.733	2.065	0.118	1.167	0.236	0.481	0.210
C9YUL1	glycosyl hydrolase	0.198	0.013	0.047	0.013	0.000	0.000	0.000	0.000
C9YUZ2	xylanase/cellulase	1.235	0.188	1.092	0.043	0.918	0.029	0.612	0.231
C9YVN3	secreted protein	0.011	0.016	0.023	0.032	0.000	0.000	0.000	0.000

C9YVP9	endo beta-1,4-xylanase	1.000	0.074	1.671	0.242	1.895	0.521	1.039	0.056
C9YYV2	arabinofuranosidase	0.382	0.125	0.304	0.236	0.157	0.111	0.098	0.028
C9Z041	arabinase	0.000	0.000	0.140	0.028	0.030	0.014	0.110	0.042
C9Z0D5	endoglucanase	0.144	0.027	0.225	0.053	0.238	0.141	0.200	0.000
C9Z1T6	lactonase	0.250	0.032	0.352	0.048	0.273	0.129	0.102	0.016
C9Z271	glycosyl hydrolase	0.056	0.079	0.019	0.026	0.000	0.000	0.000	0.000
C9Z2N2	endo-beta-1,6-galactanase	1.214	0.354	1.009	0.189	0.464	0.152	0.152	0.013
C9Z2V1	endo beta-1,4-xylanase	0.040	0.057	0.080	0.000	0.080	0.000	0.000	0.000
C9Z351	endoglucanase	0.447	0.074	0.539	0.019	0.368	0.037	0.105	0.074
C9Z4J7	alpha-N-furanosidase	0.722	0.016	0.778	0.157	0.611	0.031	0.128	0.086
C9Z507	glycosyl hydrolase	0.652	0.108	0.753	0.004	0.715	0.081	0.476	0.211
C9Z5L1	glucose / sorbosone dehydrogenase	7.163	0.548	6.550	0.141	4.513	0.654	2.750	0.389
C9Z737	galactan endo-1,6-beta-galactosidase	0.128	0.124	0.203	0.115	0.236	0.182	0.142	0.010
C9Z7A0	alpha-galactosidase	0.739	0.041	0.783	0.143	0.674	0.092	0.558	0.215
C9Z7A1	glycosyl hydrolase	0.442	0.027	0.423	0.136	0.077	0.027	0.000	0.000
C9Z804	cellulose-binding protein	0.614	0.096	0.682	0.193	0.295	0.096	0.136	0.064
C9Z9L5	cellulase B	1.987	0.453	1.462	0.218	0.692	0.073	0.718	0.036
C9Z9L6	cellulase	9.265	0.069	7.181	0.991	6.745	1.331	7.088	3.480
C9Z9L7	cellulase	14.492	1.214	11.483	0.118	8.150	1.414	7.800	2.546
C9ZB22	alpha-arabinanase	0.127	0.040	0.148	0.030	0.155	0.000	0.070	0.060
C9ZBE6	alpha-L-fucosidase	0.034	0.010	0.034	0.010	0.000	0.000	0.000	0.000
C9ZCR4	glycosyl hydrolase	0.482	0.114	0.321	0.025	0.210	0.095	0.085	0.057
C9ZD50	cellulase	1.956	0.062	2.235	0.666	1.118	0.250	0.338	0.187
C9ZD59	arabinofuranosidase	0.840	0.396	0.590	0.438	0.190	0.127	0.000	0.000
C9ZE94	arabinofuranosidase	0.311	0.253	0.189	0.160	0.019	0.027	0.000	0.000

C9ZE95	xylanase A	9.923	1.115	10.298	4.909	5.385	2.312	1.779	0.802
C9ZEP9	cellulase	6.217	0.306	8.050	0.000	6.008	1.190	3.825	1.968
C9ZEQ0	cellulase	9.039	1.081	8.088	1.262	7.809	2.683	5.152	2.863
C9ZFW2	beta-xylosidase	0.796	0.079	0.704	0.052	0.250	0.092	0.046	0.065
C9ZFW3	arabinofuranosidase	1.943	0.027	2.019	0.080	1.434	0.133	0.962	0.187
C9YTD0	endoglucanase	0.446	0.227	0.268	0.025	0.250	0.101	0.000	0.000
C9YU67	pectate lyase	0.486	0.000	0.459	0.038	0.068	0.057	0.000	0.000
C9YYV4	acetyl-xylan esterase	0.184	0.037	0.105	0.000	0.000	0.000	0.000	0.000
C9Z2V2	acetyl-xylan esterase	0.000	0.000	0.000	0.000	0.042	0.059	0.000	0.000
C9Z8E5	chitinase C	0.125	0.022	0.094	0.066	0.117	0.077	0.016	0.022
C9ZAD0	carbohydrate-binding protein	0.111	0.157	0.078	0.110	0.000	0.000	0.000	0.000
C9ZEQ1	cellulase	3.070	0.236	3.478	0.114	3.952	0.722	3.871	1.718
C9ZGN8	chitinase A	0.217	0.024	0.158	0.012	0.133	0.000	0.100	0.047
C9YVP7	feruloyl esterase	0.000	0.000	0.157	0.020	0.100	0.061	0.000	0.000
C9ZE96	feruloyl esterase	0.688	0.236	0.635	0.044	0.292	0.118	0.198	0.103
Cell wall, membrane or envelope biogenesis									
C9Z1V1	carbohydrate isomerase	0.140	0.000	0.058	0.082	0.012	0.016	0.035	0.016
C9Z2W0	glycosyl hydrolase	0.022	0.031	0.000	0.000	0.033	0.015	0.000	0.000
C9Z8V2	D-alanyl-D-alanine carboxypeptidase	0.067	0.000	0.117	0.071	0.167	0.141	0.083	0.024
C9YWP7	lytic transglycosylase	0.143	0.112	0.183	0.034	0.024	0.034	0.008	0.011
C9Z058	N-acetylmuramoyl-L-alanine amidase	0.530	0.011	0.567	0.021	0.448	0.000	0.224	0.063
Defense mechanism									
C9Z7C3	peptidase	0.013	0.018	0.088	0.053	0.088	0.053	0.025	0.035
C9Z7C8	superoxide dismutase	0.375	0.059	0.229	0.147	0.229	0.088	0.354	0.029

Differentiation and secondary metabolism									
C9YYT5	aminohydrolase	0.000	0.000	0.000	0.000	0.009	0.012	0.026	0.025
C9YU79	hydroxylase SgaA-N like protein	0.000	0.000	0.000	0.000	0.000	0.000	0.019	0.026
C9Z3A4	spore-associated protein	0.000	0.000	0.143	0.202	0.107	0.152	0.179	0.253
C9ZD97	BldKB-like	0.077	0.022	0.338	0.152	0.454	0.250	0.138	0.065
Energy production and conversion									
C9Z2U3	aldehyde dehydrogenase	0.000	0.000	0.000	0.000	0.000	0.000	0.032	0.045
C9YXV0	NuoM, NADH dehydrogenase subunit	0.000	0.000	0.000	0.000	0.018	0.025	0.027	0.013
C9ZE86	cytokinin dehydrogenase	0.123	0.013	0.132	0.053	0.170	0.080	0.113	0.000
C9YTU0	quinol oxidase	0.000	0.000	0.014	0.019	0.027	0.038	0.000	0.000
Inorganic ion metabolism									
C9YVE4	alkaline phosphatase	0.000	0.000	0.018	0.025	0.009	0.013	0.045	0.063
C9YVU0	phosphatase	0.000	0.000	0.010	0.014	0.020	0.029	0.000	0.000
Lipid metabolism									
C9YTK3	lipase	0.158	0.074	0.430	0.261	0.561	0.273	0.325	0.112
C9ZCR8	Sub1 cutinase/suberinase	0.000	0.000	0.214	0.168	0.333	0.202	0.333	0.269
C9YUN4	cholesterol esterase	0.043	0.000	0.152	0.154	0.174	0.061	0.130	0.000
C9Z1F6	lipase	0.317	0.165	0.550	0.448	0.717	0.024	0.550	0.212
C9Z4H0	3 hydroxyacyl coA reductase	0.000	0.000	0.074	0.052	0.574	0.131	0.593	0.052
C9Z5Z2	glycerophosphoryl diester phosphodiesterase	0.140	0.000	0.221	0.016	0.233	0.132	0.174	0.049
C9Z707	FadA acetyl-coA C-acetyltransferase	0.000	0.000	0.000	0.000	0.000	0.000	0.037	0.052
C9ZCR0	sphingolipid ceramide N-deacylase	0.020	0.028	0.020	0.028	0.047	0.009	0.013	0.019
C9Z6Y6	cholesterol esterase	0.783	0.123	1.043	0.738	1.522	0.246	1.109	0.338

C9ZG71	esterase A	0.500	0.263	0.286	0.040	0.186	0.020	0.029	0.040
C9Z8A2	phospholipase C	0.021	0.010	0.068	0.019	0.116	0.126	0.041	0.000
Posttranslational modification, protein turnover									
C9Z0U4	ATP-dependent zinc metalloprotease	0.000	0.000	0.014	0.020	0.028	0.040	0.063	0.030
Signal transduction									
C9YSZ5	two component sensor kinase	0.000	0.000	0.000	0.000	0.008	0.011	0.000	0.000
Transport, secretion, efflux									
C9YYU9	substrate-binding component of ABC transporter	0.011	0.015	0.000	0.000	0.021	0.030	0.000	0.000
C9Z307	glycerol uptake facilitator protein	0.000	0.000	0.000	0.000	0.037	0.052	0.074	0.105
C9ZEJ9	carbohydrate binding lipoprotein	0.000	0.000	0.011	0.016	0.000	0.000	0.000	0.000
C9YYE4	solute-binding protein	1.223	0.346	1.074	0.165	0.702	0.150	0.266	0.135
C9Z3N2	ABC-type thiamine transport system	0.026	0.036	0.038	0.054	0.013	0.018	0.000	0.000
C9YT14	solute-binding lipoprotein	0.265	0.125	0.324	0.166	0.471	0.083	0.309	0.021
C9YZP9	solute-binding protein	0.367	0.094	0.333	0.047	0.250	0.024	0.233	0.047
C9Z451	cellobiose-binding transport system	0.625	0.324	0.427	0.162	0.604	0.177	0.229	0.029
C9ZDW4	ABC-type xylose transport system	0.575	0.106	0.200	0.106	0.050	0.000	0.000	0.000
C9YUG2	ABC-type carbohydrate transport system protein	1.686	0.687	2.043	1.111	1.671	0.303	1.414	0.020
C9YUK3	aliphatic sulfonate ABC transporter	1.692	0.036	1.795	0.036	1.385	0.073	1.333	0.218
C9YVX8	xylose ABC transporter	0.579	0.409	0.579	0.037	0.658	0.186	0.434	0.056
C9YWP0	branched-chain amino acid ABC transporter	5.267	0.576	8.523	1.036	8.279	0.756	5.558	2.138
C9YXA8	glycine betaine-binding lipoprotein	4.861	0.589	8.569	0.295	7.056	0.079	4.792	1.041
C9Z204	glutamate uptake system	1.621	0.439	1.690	0.683	2.172	0.390	2.121	0.366

C9Z473	DesF, iron transport system	0.000	0.000	0.041	0.057	0.054	0.038	0.027	0.038
C9Z5D4	oligopeptide-binding transport system	0.785	0.261	0.400	0.196	0.477	0.174	0.238	0.033
C9ZBY7	transport system peptide-binding protein	0.017	0.024	0.051	0.000	0.025	0.012	0.110	0.012
C9ZFJ5	high-affinity phosphate-binding protein	23.590	3.445	30.103	2.828	26.090	2.230	15.218	3.536
C9ZHD5	maltose-binding protein	1.167	0.236	0.644	0.094	0.656	0.110	0.389	0.173
C9ZD81	carbohydrate binding protein	0.189	0.000	0.176	0.019	0.162	0.000	0.068	0.019
General function prediction only									
C9YSY7	acyltransferase	0.018	0.025	0.071	0.101	0.054	0.025	0.036	0.051
C9Z047	amidase	0.006	0.008	0.029	0.042	0.012	0.000	0.000	0.000
C9ZCL9	tripeptidylaminopeptidase	0.132	0.087	0.325	0.335	0.518	0.211	0.114	0.112
C9YT01	hydroxylase	0.000	0.000	0.000	0.000	0.000	0.000	0.017	0.024
C9Z790	phosphohydrolase	0.050	0.071	0.000	0.000	0.200	0.071	1.250	0.141
C9ZGH4	beta lactamase	0.022	0.031	0.022	0.031	0.043	0.061	0.217	0.061
C9ZGK7	alpha-N-acetylglucosaminidase	0.012	0.017	0.025	0.035	0.049	0.035	0.012	0.017
C9Z871	oxidoreductase	0.135	0.038	0.216	0.229	0.459	0.000	0.189	0.038
C9ZAW6	aminopeptidase	0.278	0.052	0.176	0.013	0.074	0.052	0.037	0.000
C9YT92	lipoprotein	0.689	0.134	0.703	0.229	0.959	0.096	1.176	0.210
C9YWG0	penicillin acylase	0.000	0.000	0.000	0.000	0.005	0.007	0.005	0.007
C9YXR8	aminopeptidase	0.706	0.416	0.382	0.166	0.294	0.042	0.206	0.042
C9ZGP4	zinc-binding carboxypeptidase	0.190	0.042	0.140	0.085	0.000	0.000	0.000	0.000
C9ZGS4	galactose oxidase	0.404	0.016	0.303	0.016	0.213	0.016	0.079	0.095
C9ZBK5	phosphoesterase	0.016	0.023	0.033	0.046	0.172	0.058	0.098	0.046
C9YV55	phosphoesterase	0.000	0.000	0.000	0.000	0.000	0.000	0.018	0.025
Function unknown									

C9Z6Q2	0.038	0.000	0.000	0.000	0.000	0.000	0.000	0.000
C9Z2P4	0.000	0.000	0.000	0.000	0.007	0.010	0.000	0.000
C9ZDW5	0.000	0.000	0.014	0.020	0.000	0.000	0.014	0.020
C9YTI6	0.034	0.049	0.000	0.000	0.103	0.146	0.034	0.000
C9Z4C3	0.688	0.265	0.500	0.177	0.188	0.088	0.125	0.177
C9Z884	0.000	0.000	0.006	0.008	0.000	0.000	0.000	0.000
C9YUE9	0.095	0.067	0.429	0.202	0.476	0.337	0.810	0.135
C9Z4B7	0.079	0.037	0.053	0.074	0.079	0.037	0.132	0.112
C9Z5Y6	0.017	0.024	0.000	0.000	0.069	0.098	0.000	0.000
C9YUN3	0.045	0.064	0.045	0.064	0.045	0.064	0.000	0.000
C9YVL7	0.013	0.019	0.092	0.130	0.105	0.000	0.000	0.000
C9Z4J0	0.351	0.015	0.532	0.030	0.394	0.075	0.351	0.075
C9Z7P8	0.040	0.057	0.000	0.000	0.000	0.000	0.000	0.000
C9ZGQ1	0.500	0.479	0.323	0.182	0.145	0.068	0.065	0.000
C9YTI5	0.000	0.000	0.000	0.000	0.056	0.079	0.000	0.000
C9YUA5	0.000	0.000	0.000	0.000	0.025	0.035	0.025	0.035
C9YV41	0.607	0.000	0.518	0.025	0.446	0.076	0.250	0.152
C9YYN8	0.367	0.047	0.267	0.000	0.000	0.000	0.000	0.000
C9Z064	0.139	0.118	0.278	0.157	0.222	0.079	0.222	0.079
C9Z2Q2	0.250	0.096	0.159	0.096	0.000	0.000	0.000	0.000
C9Z3S4	0.088	0.000	0.000	0.000	0.059	0.000	0.221	0.062
C9Z4L1	0.000	0.000	0.012	0.017	0.083	0.051	0.143	0.168
C9Z760	0.347	0.084	0.178	0.036	0.119	0.000	0.093	0.012
C9Z770	0.024	0.011	0.127	0.090	0.135	0.191	0.063	0.067
C9Z7D6	0.154	0.000	0.212	0.136	0.212	0.082	0.154	0.000

C9Z8L7	0.000	0.000	0.105	0.149	0.158	0.223	0.079	0.037
C9Z8N7	0.125	0.044	0.063	0.000	0.063	0.000	0.109	0.066
C9Z9T4	0.045	0.000	0.114	0.032	0.000	0.000	0.000	0.000
C9ZCQ1	0.092	0.130	0.013	0.019	0.000	0.000	0.000	0.000
C9ZDY2	0.000	0.000	0.000	0.000	0.032	0.045	0.000	0.000
C9ZHF3	0.125	0.025	0.107	0.051	0.027	0.038	0.080	0.038
**Total**	144.708	1.970	153.719	2.680	127.140	15.400	89.015	19.700

Supplementary Table S2 Abundance of *Streptomyces scabiei* extracellular proteins associated with the functional group Carbohydrate metabolism class after 10 to 60 days of incubation

	Normalized spectral abundance factor (relative abundance (%) within the Carbohydrate metabolism class)			
CAZy group	Day 10	Day 20	Day 30	Day 60
Carbohydrate esterases	1.34 (2.20)	1.13 (2.36)	0.82 (1.66)	0.50 (1.02)
Polysaccharide lyases	2.70 (4.39)	1.82 (3.30)	0.65 (1.32)	0.22 (0.45)
Glycosyl hydrolases	50.95 (82.79)	45.99 (83.54)	42.55 (86.22)	43.13 (88.20)
Cellulases and endoglucanases	34.85 (56.63)	30.14 (54.75)	29.83 (60.45)	34.23 (70.00)
Unclassified proteins	6.55 (10.62)	6.11 (10.80)	7.33 (11.80)	5.05 (10.33)
